# Diabetes and Quality of Life: A Ugandan Perspective

**DOI:** 10.1155/2014/402012

**Published:** 2014-03-02

**Authors:** Ronald Nyanzi, Robert Wamala, Leonard K. Atuhaire

**Affiliations:** Makerere University, Kampala, Uganda

## Abstract

Quality of life of diabetic patients is not a new concept in literature. The contentious issue however is whether factors associated in literature with quality of life apply to diabetic patients in Uganda. A sample of 219 outpatients attending Mulago diabetic clinic—a national referral hospital in Uganda—is used to provide an understanding of this issue. Quality of life is assessed in the dimensions of role limitation due to physical health, emotional health, treatment satisfaction, physical endurance, and diet satisfaction based on a five-point Likert scale. The analysis is made by patients' characteristics, medical conditions, lifestyle factors, and type of medication using frequency distributions, summary statistics, and a Poisson regression. In the results, we confirm a consensus regarding the influence of age and education level on the quality of life in the dimensions of role limitation and physical endurance (P < 0.05). A similar conclusion is reached with regards to impact of diabetic foot ulcers in the dimension of physical endurance. Thus, the factors associated with quality of life are not entirely unique to diabetic patients in the country.

## 1. Introduction

Diabetes, a major noncommunicable disease, remains highly prevalent with an increasing incidence globally. It is classified under three major groups, namely, type 1, type 2, and gestational diabetes [1]. According to the International Diabetes Federation, type 2 accounts for over 90% of all cases of diabetes. Globally, the number of patients with diabetes is expected to increase from 285 million to 439 million by 2030 [2]. The disease was previously thought to be rare in Africa; however, as a result of changes in the lifestyle, feeding patterns, and levels of physical activity among other factors, the prevalence has increased in many African countries over the past few decades. For example, the diabetic population in Uganda, estimated at about 98,000 in 2000, increased more than fifteen times (1.5 million) in a decade [3]. Based on the country's estimated population of 30 million people in 2010 [4], the figure implies that about five percent of the country's population was diabetic. This evidence suggests that the number of diabetic patients in the country is highly likely to maintain an upward trend.

The upward trend in the number of diabetic patients points to the need for improved treatment and care for the disease. The fact that treatment for the disease and its associated risk factors are highly complex, a considerable patient education and medical monitoring are required [5]. Thus, the patient is required to regulate blood sugars amidst required changes in lifestyle factors and the unpleasant medication that usually accompanies the disease in order to maintain a correct degree of metabolic control. The fact that these changes make the patients vulnerable to stress, their quality of life is highly bound to be affected. The predictors of quality of life of diabetic patients are identified by Imayama et al.'s [6] study as personal, medical, and lifestyle factors. Particularly, the study noted that old age, higher income, higher score on activity (personality) trait, not using insulin, having fewer comorbidities, lower body mass index (BMI), being a nonsmoker, and a higher physical activity level were significantly associated with better health related quality of life in adults with type 2 diabetes. Further, their study identifies the factors age, gender, marital status, income, activity trait, insulin, comorbidities, higher BMI, smoking, and higher general diet score with life satisfaction. Similar conclusions regarding the influence of socioeconomic status of patient, education level, and status of physical complications on quality of life are reached among types 1 and 2 diabetic patients in Nigeria [7]. These findings are supported by the literature in USA [8], Oman [9], India [10], and Singapore [11], to mention but a few. Although there may be variations with regards to the influence of these factors, the consensus is that quality of life varies by characteristics of patients, type of medication, and variations in medical conditions across patients. For example, Al-Maskari et al. [9] report a better quality of life among younger patients (below 40 years) while Trief et al.'s [8] study associates a better quality of life to older patients (above 65 years).

The evidence above demonstrates a wealth of knowledge about diabetes in these countries. However, Uganda remains with scanty or nearly no comprehensive equivalent studies about the same; a situation that is bound to affect management of the disease. In other words, is it questionable whether the factors associated with quality of life of diabetic patients in the aforementioned studies apply to the patients in Uganda. These studies however provide a basis obtaining an understanding of the factors associated with quality of life of diabetic patients in the country. Thus, our study was aimed at assessing the factors associated with quality of life among diabetic patients in the country. Particularly, the study seeks to establish how the factors in the literature related to diabetic patients in the Uganda.

## 2. Data and Methods

This study was based on a cross-sectional design. Primary data was obtained from outpatients diagnosed with diabetes at Mulago Hospital diabetic clinic using interviewer administered questionnaires. The hospital is the national referral health centre in Uganda and operates an outpatients' diabetic clinic ounce a week. It also has inpatient facilities where medical care is provided throughout the week. Both outpatients and inpatients receive free medical care, including medicines, when available in the hospital. This background makes the hospital a good location in the country for undertaking the study. However, the study population comprised of all outpatients who were expected to attend diabetic clinic days in January 2013 (*N* = 480). A sample of 219 patients was determined using Yamane's [12] sample size formulae based on a precision of 5%. The patients considered on each of the four clinic days were determined using systematic sampling. A medical register of outpatients compiled by the nursing officer in charge of each of the clinic days was adopted as a sampling frame. A sampling interval of two was used in selecting patients for interviewing. The patients were briefed by the nursing officer in change regarding the importance of the study. Thus, issues of nonresponse or refusal to participate in the study did not arise.

Particularly, the data was obtained from the selected outpatients using the quality of life standardized instrument developed by Nagpal et al. [10]. Our investigations were however based on five quality of domains, namely, role limitation due to physical health, mental health, treatment satisfaction, physical endurance, and diet satisfaction. The items or questions in the various domains were investigated based on a five-point Likert scale. The items in the various quality of life domains were tested and confirmed for reliability using Cronbach's alpha [10]. In other words, a high alpha value (*α* > 0.7) obtained for the various quality of life dimensions implies reliability of the items adopted in assessing the latent constructs.

In the analysis, summated scores or indices were generated for each of the five domains [13, 14]. The summated indices in the various domains comprise of nonnegative integer values. In addition to quality of life, data on patient's characteristics (age, sex, and education), lifestyle factors (status of smoking and alcohol consumption), medical conditions (duration with diabetes, type of diabetes, and occurrence of diabetic complication), and methods of medication (insulin therapy, oral therapy, or combination of the two) was compiled. The analysis was done at three stages: first, a descriptive summary of patient's characteristics and their quality of life were made using summary statistics. Second, a bivariate assessment of quality of life domains by patients' characteristics, medical conditions, lifestyle factors, and methods of medication was made using a Kruskal-Wallis test, a nonparametric test. Choice of the approach was based on the fact that the summated scores were obtained from ordinal responses. The outcomes on each of the five domains do not represent real values; they were considered as ranks. Otherwise, an analysis of variance (ANOVA) parametric alternative would be the appropriate tool for investigating the data. All variables that yielded relatively small probability values (*P* < 0.5) in the assessment were considered for further analysis at the multivariable stage. Third, the net-impact of these variables on quality of life was investigated at a multivariable analysis using a Poisson regression. The fitted model was investigated for appropriateness when compared to the Negative binomial alternative using the Pearson chi-square goodness-of-fit test [15] (Paternoster & Brane, 1997). The diagnostic test was focused on assessing the overdispersion aspect in the fitted Poisson regression model. Associations between the dependent and independent variables were established at 5% and 1% levels, unless otherwise stated.

## 3. Results

Tables 1–3 present a descriptive summary of patients' characteristics, medical conditions, and methods of medication as well as life style factors. A summary of these variables is made subsequently.

The characteristics of diabetic patients according to Table 1 can be summarized as follows: predominantly female (72.6%) and above 49 years of age (56.1%); the highest proportion had primary education (47.5%), followed by 36.5% with postprimary education while the rest had no formal education.

With regards to medical conditions in Table 2, about four in every nine (44.8%) were type 1 diabetic patients while slightly less than five in every nine (53.9%) reported having diabetic foot ulcers; majority reported having retinopathy (66.2%) and hypertension (74.4%). Further, majority of patients were on oral therapy (66.2%), followed by 36.1% on insulin therapy while the rest were on a combination of the two.

In the results of lifestyle factors according to Table 3, the vast majority were not engaged in smoking (98.2%) and alcohol consumption (89.5%).

### 3.1. Quality of Life

As earlier indicated, quality of life was assessed in this study based on five domains, namely, role limitation due to physical health, mental health, treatment satisfaction, physical endurance, and diet satisfaction.  Table 4 presents summary statistics on these domains.

According to Table 4, a mean estimate of 4.04 implies minimal role limitation experienced with regards to physical health. However, lower rating with regards to efficiency at work and absence from work and travelling (business tour, holiday, and general outings) demonstrates difficulties in these aspects. Overall mean estimates of 3.82 and 3.96 demonstrate that the patients were satisfied with their mental health situation and treatment received, respectively. Estimates of 3.83 and 3.86 in the dimensions of physical endurance and diet satisfaction, respectively, demonstrate that patients were sometimes affected in the aforementioned aspects.

### 3.2. Differentials in Quality of Life

Differentials in the quality of life were assessed at the bivariate analysis by patients' characteristics, lifestyle factors, medical conditions, and methods of medication.  Table 5 presents a bivariate assessment of quality of life by the factors using a Kruskal-Wallis test. As earlier stated, the assessment at the bivariate level is geared towards identifying variables to be considered for further analysis in the multivariable stage.

In the assessment according to Table 5, the following can be deduced: quality of life in the domain of role limitation varied significantly by age, education level, status of diabetic foot ulcers, retinopathy, and alcohol consumption (*P* < 0.05). Further, the variables that yielded small probability values (*P* < 0.5) in the assessment were sex and type of diabetic treatment.

In the domain of emotional health, quality of life varied significantly by only status of hypertension (*P* < 0.05). The variables that yielded small probability values in the domain where age, education level, status of diabetic foot ulcers, and alcohol consumption (*P* < 0.5).

Significant variations in the domain of treatment satisfaction were observed only by status of hypertension (*P* < 0.05). Further, variables that yielded small probability values in this domain were sex, education level, type of diabetes, status of diabetic foot ulcers, smoking, and alcohol consumption as well as type of diabetic treatment.

In the domain of physical endurance, significant variations were noted by age, sex, education level, status of diabetic foot ulcers, and retinopathy (*P* < 0.01). With the exception of status of smoking, the rest of the variables yielded small probability values in the assessment (*P* < 0.5).

Status of foot ulcers and retinopathy were the only variables that varied significantly with quality of life in the domain of diet satisfaction (*P* < 0.01). The variables that yielded small probability values with quality of life in the assessment were sex, age, education level, and type of diabetes as well as status of hypertension and smoking (*P* < 0.5).

Further analysis at the multivariable stage was undertaken in the domains of role limitation and physical endurance where a considerable number of variables were noted to be significantly associated with quality of life. Thus, Table 6 presents a multivariable analysis based on the Poisson regression in these dimensions. The table presents exponentiated coefficients (MR), standard errors (Std. Err), and probability values (*P* values). The IRR represents the change in the dependent variable in terms of a percentage increase or decrease. The precise percentage is determined by the amount the IRR is either above or below 1.

### 3.3. Regression Diagnostics

Table 7 presents results of the chi-square goodness-of-fit test for the fitted Poisson regression models in the domains of role limitation and physical endurance; a summary of the results is made subsequently.

Results of the Pearson goodness-of-fit test in Table 6 indicate that the distribution of quality of life in the domains of role limitation and physical endurance does not significantly differ from a Poisson distribution (*P* > 0.05). This implies that the assumptions of adopting the Poisson model were supported.

### 3.4. Summary of the Results

The predictors of quality of life in the dimensions of role limitation were patient's age, education level, and status of diabetic foot ulcers (*P* < 0.05). Similar variables were associated with quality of life in the dimension of physical endurance (*P* < 0.05). The findings with regards to the influence of these variables can be summarized as follows.

- Quality of life was approximately 13% (MR = 0.87) and 18% (MR = 0.82) lower for diabetic patients above 59 years of age when compared to those below 50 years in the domains of role limitation and physical endurance, respectively.
- Quality of life was about 16% (MR = 1.16) and 19% (MR = 1.19) higher in the domain of role limitation among patients with secondary and tertiary education, respectively, when compared to those with no education. Likewise, quality of life was about 11% (MR = 1.11) and 16% (MR = 1.16) higher among patients with secondary and those with tertiary education in the domain of physical endurance when compared to those with no education.
- Quality of life was about 8% (MR = 0.92) lower in the domain of physical endurance among patients with diabetic foot ulcers when compared to those without the condition. No significant impact of status of diabetic foot ulcers on quality of life was noted in the dimension of role limitation (*P* < 0.05).

Worth noting is that quality of life of diabetic patients did not vary significantly by gender, status of smoking and alcohol consumption, treatment therapy, and type of diabetes as well as prevalence of hypertension and retinopathy (*P* > 0.05).

## 4. Discussion

Factors associated with quality of life are certainly not entirely unique to diabetic patients in Uganda. In our results, quality of life among diabetic patients in the country is associated with age, education level, and status of diabetic foot ulcers. Particularly, quality of life is higher among diabetic patients at lower ages (below 50 years), those with secondary and tertiary education, and patients without diabetic foot ulcers. With regards to age, the findings in our study are in agreement with studies elsewhere that associate better quality of life with younger patients [5, 6, 8, 9]. The consensus in these studies is that quality of life is higher among patients in the lower ages. On the contrary, Trief et al. [8] study reported better quality of life among diabetic patients at latter ages (above 64 year) compared to the younger counterparts (30–64).

According to our findings, the minimum level of education associated with better quality of life among diabetic patients in Uganda is secondary. One could as well conclude that the minimum level of education associated with better quality of life in the dimension of role limitation is primary (*P* < 0.1). Thus, an argument of the minimum education level of patients that registered poor quality of life being low [7] is supported. All the same, literature that identifies education level of patients as a predictor of quality of life of diabetic patients (e.g., [7, 11, 16]) is highly supported. Better quality of life among patients with higher education is attributed to the fact that they can easily read and understand the effects of diabetes on their health; thus, they are more likely to adjust to their recommended treatment and diet regimen [7]. The argument in their study is that education is an essential factor in understanding self-care management of diabetes, glycaemic control, and perception of self-worth.

Although Issa and Baiyewu's [7] study reveals better quality of life of type 2 diabetic patients in Nigeria, no significant variations were noted among the patients in Uganda. This evidence demonstrates variations in quality of life of diabetic patients across countries and/or regions. Regarding status of foot ulcers, this study confirms literature that identifies that variable as a predictor of quality of life [17–19] particularly in the dimensions of physical health and functioning. The consensus is that patients without diabetic foot ulcers have better quality of life with regards to physical functioning and health. The influence of diabetic foot ulcers on quality of life is attributed mainly to the pain and mobility problems that usually result from the condition [20]. With regards to status of retinopathy andhypertension, our study does not associate the medical conditions with quality of life of diabetic patients in Uganda. This evidence is contrary to a study among patients in the USA that associated hypertension among diabetic patients with lower quality of life [21].

In Uganda, our study reveals that lifestyle factors (i.e., smoking and alcoholic consumption) were not significant predictors of quality of life of diabetic patients. These findings are contrary to studies that associate smoking to poor quality of life of diabetic patients in all dimensions [22, 23]. Likewise, the findings in Uganda are contrary to Altenburg et al.'s [24] study on the effect of alcohol consumption of diabetic patients in Germany. Their study revealed that higher lifestyle alcohol consumption is associated with development of diabetic foot ulcers, a condition that is certainly proven to lower the quality of life of the patients.

There is no consensus in the literature regarding the influence of medication methods (insulin therapy, oral, and insulin and oral therapy) on quality of life of diabetic patients. In USA, insulin use is associated with decreased health related quality of life of type 2 diabetic patients [25]. On the contrary, Akinci et al.'s [26] study revealed that insulin use was associated with better health related quality of life of diabetic patients in Turkey. In Israel, patients on oral therapy had a better quality of life compared to those who were using insulin [27]. In Uganda, the type of treatment was not significantly associated with the quality of life of diabetic patients. The nonsignificant influence of type of medication on quality of life of diabetic patients in Uganda supports the argument of mixed conclusion reached by whether or not insulin is administered. Funnell [28] however asserts that insulin therapy is required to maintain treatment targets although patients' concerns about its effect on their quality of life may be legitimate. On affirming the application of insulin therapy, Funnell [28] argues that that providers need to provide continuous information and support to address the barriers associated with the medication.

In summary, our findings demonstrate a consensus regarding the influence of age, education level, and status of foot ulcers on the quality of life of diabetic patients across countries. However, this conclusion may not be entirely applied to diabetic patients in other countries elsewhere. In addition, our findings are limited in explaining variations (if any) in factors associated with quality of life between types 1 and 2 diabetic patients although the study controls this aspect in the investigations. Thus, the aspect must be put into consideration in the interpretation of our findings.

## Figures and Tables

**Table 1:** Distribution of diabetic patients' characteristics.

Characteristics	*n*	Percentage (%)
Sex		
Male	60	27.4
Female	159	72.6
Total	**219**	**100.0**
Education level		
No education	35	16.0
Primary	104	47.5
Secondary	58	26.5
Tertiary	22	10.0
Total	**219**	**100.0**
Body mass Index		
<20	24	11.0
20–24.9	95	43.4
25–29.9	73	33.3
>30	27	12.3
Total	**219**	**100.0**
Age		
Below 50	94	42.9
50–59	64	29.2
Above 59	61	27.9
Total	**219**	**100.0**

**Table 2:** Distribution by medical conditions and methods of medication.

Variables	*n*	Percentage (%)
Diabetes type		
Type 1	98	44.8
Type 2	121	55.2
Total	**219**	**100.0**
Diabetic foot		
No	101	46.1
Yes	118	53.9
Total	**219**	**100.0**
Retinopathy		
No	145	66.2
Yes	74	33.8
Total	**219**	**100.0**
Hypertension		
No	163	74.4
Yes	56	25.6
Total	**219**	**100.0**
Diabetes treatment		
Insulin therapy	79	36.1
Oral therapy	134	66.2
Insulin and oral	6	2.7
Total	**219**	**100.0**

**Table 3:** Distribution by life style factors.

Variables	*n*	Percentage (%)
Smoking status		
No	215	98.2
Yes	4	1.8
Total	**219**	**100.0**
Alcoholic status		
No	196	89.5
Yes	23	10.5
Total	**219**	**100.0**

**Table 4:** Summary statistics on quality of life.

Domains	*n*	Items^a^	Mean^b^	Std. Dev	Min	Max
Role limitation	219	6	24.2 (4.04)	4.61	6	30
Emotional/mental health	219	5	19.1 (3.82)	2.84	11	25
Treatment satisfaction	219	4	15.8 (3.96)	1.72	9	20
Physical endurance	219	5	19.2 (3.83)	3.96	8	25
Diet satisfaction	219	3	11.6 (3.86)	1.25	6	15

Note: summary statistics are based on summated scales.

^a^denotes number of questions in a domain.

^b^denotes mean values based on summated and average scores.

**Table 5:** Differential in quality of life in a bivariate analysis.

Independent variables	Quality of Life domains (average rank)^a^				
	Role limitation	Emotional health	Treatment satisfaction	Physical endurance	Diet satisfaction
Sex					
Male	123.2	107.3	116.9	135.6	117.0
Female	105.0	111.0	107.4	100.3	107.4
	χ^2^ = 3.6, *P* = 0.058	χ^2^ = 0.1, *P* = 0.703	χ^2^ = 1.0, *P* = 0.325	χ^2^ = 13.6, *P* = 0.000	χ^2^ = 1.0, *P* = 0.316
Age group					
Below 50 yrs	127.0	105.9	111.2	136.4	12.1
50–59	118.2	118.8	120.0	110.7	108.9
Above 59 yrs	75.1	107.1	97.6	68.5	98.1
	χ^2^ = 26.4, *P* = 0.000	χ^2^ = 1.8, *P* = 0.412	χ^2^ = 4.0, *P* = 0.823	χ^2^ = 42.4, *P* = 0.000	χ^2^ = 3.9, *P* = 0.145
Educational level					
No education	74.2	86.9	95.6	76.1	96.0
Primary	100.2	113.2	105.7	99.4	105.7
Secondary	136.1	118.9	128.8	135.1	116.5
Tertiary	144.6	108.3	103.2	148.1	135.5
	χ^2^ = 30.1, *P* = 0.000	χ^2^ = 6.1, *P* = 0.107	χ^2^ = 8.0, *P* = 0.055	χ^2^ = 31.0, *P* = 0.000	χ^2^ = 6.4, *P* = 0.10
Body mass Index					
<20	115.6	107.3	100.9	124.3	117.9
20–24.9	105.8	114.4	110.0	106.1	107.2
25–29.9	108.4	105.7	114.3	106.4	112.3
>30	124.1	108.7	106.4	120.7	106.9
	χ^2^ = 2.1, *P* = 0.573	χ^2^ = 0.8, *P* = 0.838	χ^2^ = 0.9, *P* = 0.823	χ^2^ = 2.6, *P* = 0.452	χ^2^ = 0.7, *P* = 0.855
Diabetes type					
Type 1	109.3	109.9	104.2	113.9	106.6
Type 2	110.6	110.1	114.7	116.8	112.7
	χ^2^ = 0.03, *P* = 0.8755	χ^2^ = 0.001, *P* = 0.9811	χ^2^ = 1.4, *P* = 0.2223	χ^2^ = 0.67, *P* = 0.4126	χ^2^ = 0.49, *P* = 0.4804
Diabetic foot					
No	123.2	103.3	106.5	128.2	125.8
Yes	98.7	115.7	113.0	94.5	96.5
	χ^2^ = 8.1, *P* = 0.0043	χ^2^ = 2.1, *P* = 0.1481	χ^2^ = 0.55, *P* = 0.4547	χ^2^ = 15.39, *P* = 0.0001	χ^2^ = 11.63, *P* = 0.006
Retinopathy					
No	116.3	108.9	110.3	119.1	118.8
Yes	97.6	112.1	109.4	92.1	92.8
	χ^2^ = 4.3, *P* = 0.0388	χ^2^ = 0.13, *P* = 0.7208	χ^2^ = 0.01, *P* = 0.9173	χ^2^ = 8.911, *P* = 0.0028	χ^2^ = 8.27, *P* = 0.004
Hypertension					
No	110.1	102.0	113.6	112.0	112.1
Yes	109.8	133.2	128.6	104.2	104.0
	χ^2^ = 0.001, *P* = 0.9782	χ^2^ = 10.1, *P* = 0.0015	χ^2^ = 6.48, *P* = 0.0109	χ^2^ = 0.62, *P* = 0.4291	χ^2^ = 0.86, *P* = 0.4094
Diabetes treatment					
Insulin therapy	114.5	105.9	103.1	119.1	111.0
Oral therapy	109.2	111.7	114.3	106.4	110.1
Insulin and Oral	68.8	127.3	104.3	70.2	94.6
	χ^2^ = 2.9, *P* = 0.2277	χ^2^ = 0.87, *P* = 0.6462	χ^2^ = 1.60, *P* = 0.4483	χ^2^ = 4.42, *P* = 0.1097	χ^2^ = 0.375, *P* = 0.8292
Smoking status					
No	109.7	109.9	110.6	109.7	110.9
Yes	123.5	117.8	76.1	124.1	63.3
	χ^2^ = 0.18, *P* = 0.667	χ^2^ = 0.06, *P* = 0.8050	χ^2^ = 1.17, *P* = 0.2805	χ^2^ = 0.20, *P* = 0.6527	χ^2^ = 2.218, *P* = 0.1364
Alcoholic status					
No	106.4	108.5	108.1	107.5	109.8
Yes	140.5	122.8	125.8	131.7	111.5
	χ^2^ = 5.93, *P* = 0.0148	χ^2^ = 1.04, *P* = 0.3065	χ^2^ = 1.59, *P* = 0.2061	χ^2^ = 3.01, *P* = 0.0829	χ^2^ = 0.01, *P* = 0.9031

Note: assessment is based on summated scores in the various domains.

^a^Average rank is a quotient of “Rank Sum” by number of observations in a group.

**Table 6:** Regression estimates of quality of life in a multivariable analysis.

	Quality of life Dimensions					
	Role limitation			Physical Endurance		
	MR^a^	Std. Err	*P* value	MR	Std. Err	*P* value
Sex						
Male	—	—	—	—	—	—
Female	1.018	0.034	0.605	0.952	0.035	0.185
Age group						
Below 50 yrs	—	—	—	—	—	—
50–59	0.968	0.0328	0.336	0.9295	0.036	**0.056**
Above 59 yrs	0.866	0.0329	**0.000**	0.8194	0.035	**0.000**
Educational level						
No education	—	—	—	—	—	—
Primary	1.083	0.045	**0.056**	1.067	0.050	0.170
Secondary	1.160	0.055	**0.002**	1.119	0.059	**0.034**
Tertiary	1.193	0.070	**0.002**	1.169	0.076	**0.017**
Diabetes type						
Type 1	—	—	—	—	—	—
Type 2	1.022	0.050	0.649	1.015	0.031	0.787
Diabetic foot						
No		—	—	—	—	—
Yes	0.962	0.029	0.203	0.927	0.031	**0.025**
Retinopathy						
No		—	—	—	—	—
Yes	1.053	0.037	0.138	1.041	0.041	0.305
Hypertension						
No		—	—	—	—	—
Yes	0.890	0.092	0.261	0.926	0.106	0.499
Diabetes treatment						
Insulin therapy		—	—	—	—	—
Oral therapy	1.027	0.053	0.605	1.041	0.059	0.817
Insulin and oral	0.935	0.090	0.481	0.867	0.050	0.231
Smoking status						
No		—	—	—	—	
Yes	1.083	0.1161	0.455	0.984	0.1189	0.896
Alcoholic status						
No		—	—	—	—	
Yes	1.077	0.0481	**0.096**	1.065	0.054	0.210

Note: estimates are based on Poisson regression, where ^a^denotes mean ratio rate of occurrence.

**Table 7:** Assessment of goodness-of-fit of Poisson model.

Domain	χ^2^	*P*-value
Role limitation	160.9	0.9884
Physical endurance	128.0	1.0000
